# Food Sources and Nutrient Intakes of Filipino Working Adults

**DOI:** 10.3390/nu12041009

**Published:** 2020-04-06

**Authors:** Imelda Angeles-Agdeppa, Ma. Rosel S. Custodio

**Affiliations:** Food and Nutrition Research Institute, Department of Science and Technology, Bicutan, Taguig 1630, Philippines; roselcustodio@rocketmail.com

**Keywords:** working adults, usual energy intake, nutrient intake, food sources

## Abstract

Nutrition is an integral part of economic development, since it influences workers’ health and productivity. This study evaluated the usual nutrient intakes and food sources of working adults. We conducted a cross-sectional survey that involved 1264 selected working adults aged 19 to 59 years old from randomly selected job sectors. Quantitative dietary data was collected by a 2-day, non-consecutive 24 h recall, while a dietary diversity questionnaire was used to assess the types and frequency of foods consumed. Physical activity was measured using the World Health Organization global physical assessment questionnaire. The prevalence of inadequate intakes, defined as the percentage of adults with intakes less than the estimated average requirements (EAR) or acceptable macronutrient distribution ranges (AMDR) were estimated using the PC—Software for Intake Distribution Estimation (PC-SIDE) program. The mean daily energy intake of working adults was 1768 kcal/day or 74% of the Estimated Energy Requirements (EER) for this age group. The percentage contribution to the total energy of fats (58%) and proteins (34%) were excessively high. Consumption of fruits and vegetables was only 30% and 40% of the recommended nutrient intake, respectively. Salt intake was 52% above the adequate intake. Nutrient inadequacy was high in almost all nutrients, including iron (99%), folate (97.9%), riboflavin (95.8%), calcium (94.7%), vitamin C (87.3%), and thiamin (76.6%). The top five food sources of energy included rice (35.6%), pork (15.1%), fats and oils (4.7%), chicken (4.4%), and bread (3.8%). Energy and nutrient intakes of working adults is extremely sub-optimal due to the consumption of few nutrient-dense foods. This may pose a triple burden of malnutrition if left unsolved.

## 1. Background

Nutrition is a fundamental right in nation-building. The World Health Organization (WHO) has emphasized that one of the basic human rights is freedom from hunger and malnutrition, which are prerequisites for human and national development [1]. This challenge is posed for every developing nation to provide optimum nutrition to its workers who are considered the lifeblood of the economy, since nutrition influences workers’ health and productivity [2]. A recently published report by the World Bank Organization estimated that the economic costs of undernutrition in terms of lost national productivity and economic growth ranged from around 2% to 3% of the Gross Domestic Product (GDP), and up to 11% of the GDP in Africa and Asia [3]. Moreover, mortality caused by non-communicable diseases continue to rise with an average of 41 million deaths each year globally, mostly among the ages of 30 to 69 years old in low- and middle-income countries (LMIC) which are deemed as lives lost prematurely [4]. Preliminary data published by the World Economic Forum in 2011 estimated that economic output loss from NCDs, such as diabetes, heart disease, cancer, and COPD may amount to US $47 trillion in two decades [5]. Other research has also pointed out hindrances to the productivity of workers, such as obesity [6,7] and micronutrient deficiencies, such as iron deficiency anemia [8]. Furthermore, the Global Burden of Disease in 2017 reported that there was a 45.8% increase in the prevalence of diabetes as a top cause of years lived with disability in the Philippines as compared to the 2007 data. Likewise, risk factors most associated with disability and death in the Philippines are usually attributed to dietary factors (27.1%) [9].

The Philippines has experienced a rise in the prevalence of overweight/obesity from 26.6% in the 2008 National Nutrition Survey to 37.2% in 2018, reflecting a 10.6% increase over a span of 10 years among adults 20–59 years old. Anemia among women of reproductive age was 11.6%, and the prevalence of vitamin D deficiency among 20- to 39-year-old Filipino adults was 55.5% [10,11]. Unhealthy lifestyle behaviors, such as smoking (20.7%), binge drinking (55.7%), and physical inactivity (40.6%) have been defined as great challenges besetting the adult Filipino population [12,13,14].

Generally, lack of access to healthy foods is cited as one of the main barriers to a healthy diet in the workplace. It has been postulated that poor diet on the job, resulting in either undernutrition or overweight and obesity, is causing a loss in productivity of up to 20% [14]. Other risk factors include socio-demographic educational level and income or lifestyle factors, which have been associated with an unhealthy diet [15]. Since 62.1% of the Filipino population (15 years old and over) are in the labor force [16], there is a crucial need to consider programs and interventions that could improve workers’ nutritional status and healthy lifestyle practices for higher productivity and reduce the risks of adults experiencing the triple burden of malnutrition—such as under-nutrition, over-nutrition, and micronutrient deficiencies—or non-communicable diseases (NCDs).

In recent years, research has supported the role of diets to the health status of an individual, such as the risk for certain diseases. This was made possible by the existence of Food Composition Databases, which analyzed the specific nutrient content of food items. Food database systems are the basis of crafting-recommended dietary reference values [17]. However, there is a dearth of information regarding the comprehensive assessment of nutrient intakes and food sources of nutrients focused on Filipino working adults. This study aimed to evaluate the dietary intakes and food sources of working adults in randomly selected occupational sectors, and to compare nutrient intakes with the recommended levels. The results of this study could be one of the bases in crafting science-based health and nutrition policies for different job sectors.

## 2. Methodology

### 2.1. Study Design and Population

**Figure 1:**
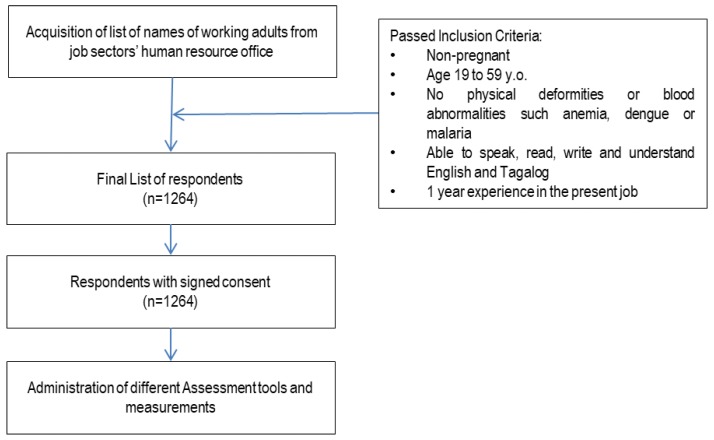
Study design.

This was a one-time, cross-sectional survey in randomly selected occupational sectors, such as business process outsourcing (BPO), factory, hospitality and foods, administration, sales, and healthcare. Participants per sector were randomly selected from the list obtained from the respective Human Resource Office. Participants included in this study were aged 19 to 59 years old, able to speak, read, write, and understand English and Tagalog. They had no physical deformities, and had at least one year’s experience in the workplace. Pregnant and lactating women were excluded from the study (Figure 1).

Study sites were the highly urbanized cities (HUC) in the three major islands of the Philippines: Luzon, Visayas, and Mindanao. These were selected from the final results of the 2012 Census of Philippine Business and Industry for All Establishments, classified under the three broad industry groups—namely, Agriculture, Industry, and Services. The total number of establishments across the country in 2012 reached *n* = 219,184. The Industry sector, with 28,221 establishments, accounted for 12.9% of the total number of establishments in 2012. According to a disaggregation by HUC, the number of business establishments are as follows: the National Capital Region (1,116 establishments), Central Visayas (161 establishments), CALABARZON (41 establishments), Central Luzon (30 establishments), and the Davao Region (24 establishments) [18]. The sample size computation (see Table 1) was computed based on the number of establishments per area, with a 95% level of confidence and 5% margin of error, using the formula below:

(1) $$n=\frac{\frac{{\left(Z\alpha \right)}^{2}}{2}\;\rho \;\left(1-\rho \right)}{MOE^{2}}$$

where Zα2 is the critical value of the normal distribution at α/2 (e.g., for a confidence level of 95%, α is 0.05 and the critical value is 1.96), *p* is the proportion employed per industry, and MOE is the margin of error.

Socio-economic and demographic characteristics or the general profile, like age, sex, educational level, income, and pre-existing medical conditions of respondents were collected using pre-tested questionnaires through a face-to-face interview.

### 2.2. Anthropometric Measurements

The height and weight of the respondents were collected using a calibrated stadiometer (207CM SECA 213 Stadiometer) and digital weighing scale (200KG SECA 874 dual display made in Germany), respectively. Height was recorded to the nearest 0.1 cm, while participants were barefoot and standing straight. Weight was recorded to the nearest 0.1 kg. Measurements of both height and weight were taken twice, and the mean was considered for analysis. Body mass index (BMI) was then calculated by dividing the weight in kilograms by the height in square meters (kg/m^2^). It was interpreted using the World Health Organization (WHO) BMI classification for adults (World Health Organization, 2006), wherein the cut-off points used in classifying the nutritional status of adults 19.0 years old and above were as follows: <18.5:Chronic Energy Deficiency/Underweight; 18.5 to 24.99—Normal; Overweight: 25.0 to 29.99; and Obese: ≥30.0.

### 2.3. Statistical Analysis for the Usual Energy Intake

Energy and nutrient intakes of the respondents were validated to identify implausible values. In the evaluation and assessment of the energy intake, the ratio of the daily energy intake and Estimated Energy Requirement (EER) of each individual was computed and then transformed to the logarithmic scale to remove outliers below −3SD and above +3SD [19]. The EER of each respondent was calculated using the Institute of Medicine (IOM) equation, considering age, sex, body weight (kg), height (m), and physical activity level (PAL).

Excessive micronutrient intakes were substituted by a random value generated from uniform distribution in the interval of the lower bound equal to the 95th percentile of the observed intake and an upper bound equal to 1.5 times the 99th percentile. Excessive micronutrient intakes were defined as those that exceeded 1.5 times the 99th percentile of the observed intake of the respondents [19]. The distribution of calcium, vitamin D, and vitamin E was highly skewed, which caused rejection of normality. Hence, a small amount of random noise ((N~ (0.35, 0.05)) was generated and added to these micronutrient intakes to adjust the intakes and rerun the analysis.

The Recommended Energy and Nutrient Intake of the Philippine Dietary Reference Intake (PDRI) 2015 was used in the evaluation of nutrient inadequacy and excessive intake of the respondents. Prevalence of inadequacy was estimated as the proportion of individuals with usual intakes below the computed average of the male and female estimated average requirement (EAR). All nutrients with EARs were assessed using the EAR cut-point method [20]. The proportion of individuals with excessive intakes was estimated using a tolerable upper intake level or upper limits per day (UL) as cut-off values.

Acceptable Macronutrient Distribution Ranges (AMDR) were used to evaluate carbohydrates, total fat, and protein intake as a percentage of energy. Nutrients with no EARs used recommended nutrient intakes (RNI) and adequate intake (AI) as a reference value, though they were not applicable to estimate the nutrients’ inadequacy. The mean intakes at or above the AI value could be assumed to have nutritionally adequate diets [21].

### 2.4. Dietary Assessment

Dietary intake of the respondents was assessed using a 2-day, non-consecutive, 24 h food recall. The 24 h food recalls of two non-consecutive days were collected using the standard form used by Food and Nutrition Research Institute (FNRI) for National Nutrition Surveys. Standard household measures were used to estimate the quantity of food consumed by the respondents. All household measures were then converted to metric weights by the trained research assistants and validated. After validation, all data were encoded in an e-template designed specifically for the study. Encoded data were again validated against the raw data by the research team. After thorough data-cleaning and validation, dietary intake was processed using a computer system called the Individual Dietary Evaluation System (IDES), developed by FNRI to describe the energy and nutrient equivalent of the food items [22]. This system makes use of the expanded Filipino Food Composition Table (FCT) containing 27 nutrients from 1359 foods.

Food group consumption was expressed by the percentage of respondents who consumed specific foods or food groups at least once on the first 24 h dietary recall regardless of the amount consumed. This method has been used in the previous studies of Siega-Riz, Deming et al. 2010, Yu, and Denney et al. 2016 [23]. The weighted percentage contribution of each food group for selected key nutrients was calculated by adding the amount of a given nutrient provided by each food group for all individuals and dividing it by the total intake of that nutrient consumed by all individuals from all foods and beverages.

### 2.5. Physical Activity

The physical activity of the adults was measured using a modified questionnaire based on activity recommendations on physical activity and health of the WHO Steps Assessment Guide. The respondents were classified as physically active or physically inactive using the questionnaire. A person not meeting any of the following criteria was considered as physically inactive or insufficiently physically active, and therefore at risk of having chronic diseases (World Health Organization STEPS Surveillance, WHO, 2002):

- Three or more days of vigorous-intensity activity for at least 20 min per day;
- Five or more days of moderate-intensity activity;
- Walking for at least 30 min per day.

### 2.6. Ethical Considerations

This study was ethically conducted in accordance with the declaration of Helsinki, guided by the Council for International Organizations of Medical Sciences’ (CIOMS) Ethical Guidelines for Biomedical Research Involving Human Subjects and the National Guidelines for Biomedical and Behavioral Research, and was approved by the DOST- FNRI Institutional Ethics Review Committee (FIERC) with registry number FIERC 2013-001. All changes (amendments) on the protocol were submitted to FIERC by the Principal Investigator (PI) for approval prior to implementation.

The participants were briefed and oriented about the objectives of the study and the data to be collected. Participation in the study was voluntary, and interested participants were asked to sign the informed consent form (ICF). They were told that they could withdraw their participation from the study at any time without any prejudice or effect on their work performance.

## 3. Results

### 3.1. Socio-Economic, Demographic, and Anthropometric Characteristics of the Study Population

The average age of the respondents was 32 years old with a range of 19–59 years. There was a similar percentage of male and female participants, and most were single (59.6%), Table 2. The majority of the respondents had either graduated from college or vocational school (63%). The participants were mostly from factory, admin, and sales sectors (66.8%); 14%, 11%, and 8% were from the Business Process Outsourcing (BPO, hospitality, and healthcare sectors, respectively. More than one-third (35%) of all the respondents had a household monthly income of Php25.000 to Php75.000. About 32% had a household monthly income of less than Php25.000 [24]. The respondents’ main source of funds was from salaries. However, around 19.3% and 3.2% of the respondents had an additional source of income from selling goods and services and money lending, respectively. A majority had been working in their current companies for 1 to 5 years. Based on BMI, almost half of the participants (46.7%) had a BMI >25, which indicates that they are overweight (30.9%) or obese (15.8%), while only 4% were underweight.

### 3.2. Energy Intake

The mean energy intake of working adult men was 1972 kcal/d, and 1566 kcal/d for women. Assuming sedentary physical activity for all participants, the energy intake of males was 18% lower compared to their Estimated Energy Requirement (EER) = 2397 kcal/d), whereas the average energy intake of females was 14% lower compared to their EER (1815 kcal/d). Supposing low active physical activity for all participants, the energy intake was far below than the EERs (24% below for males, and 23% below for females). The amounts of calories that the body needs to function while resting in 24 h (or the BMR) were almost close to the average energy intake for both sexes.

### 3.3. Nutrient Intake

The usual nutrient intakes of the respondents from the different sectors are presented in Table 3. With regard to the percentage energy distribution of carbohydrates, protein, and fat, it was seen that the respondents’ intake of these macronutrients were within the acceptable macronutrient distribution ranges (AMDR), except for the protein (15.52%), which slightly exceeded the AMDR of 10% to 15% of the total energy intake.

It was also noticeable that only 7.97 g, or about 30% to 40% of dietary fiber, compared to the recommended nutrient intake (RNI); 20 to 25 g, 33.24 mg, or a little above 50% vitamin C compared to the 65 mg RNI; and 183.52 mg, or about 82% magnesium compared to the 225 mg RNI was consumed by the respondents. Moreover, only 4.42 mg or 44.2% Vitamin E, as compared to the 10 mg adequate intake (AI) and 1617.8 mg, or 81.9% of 2000 mg potassium AI was consumed. On the other hand, the usual diet of the participants had selenium (116.84 µg), which was about 70% higher than the RNI, and sodium (1038.6 mg) that was 51.9% higher than the AI (Table 3).

The prevalence of an inadequate intake of protein was only 24%, while as a percentage of the total energy, it indicated that there was no inadequacy of protein among working adults. Also, there was no inadequacy of total fat as a percentage of total energy. However, it was noticeable that there was an excessive intake of total fat (34%) and protein (58%) as a percentage of the total energy, while a considerably high percentage of inadequacy of carbohydrates (48%) occurred.

In Table 4, inadequate and excessive intakes are shown. A high prevalence of inadequate intakes was found for iron (99%), folate (98%), riboflavin (96%), calcium (94%), vitamin c (87%), and thiamin (76%), followed by vitamin A retinol equivalent RE (40%). Niacin, vitamin B6, vitamin B12, phosphorus, zinc, and selenium intakes were adequate among working adults. The whole distribution of fiber intake did not meet the range of the recommended fiber intake (RNI: 20–25 g). The mean levels of vitamin D (4 µg/d), vitamin E (4.4 mgα−TE), sodium (1039 mg/d), and potassium (1618 mg/d) were far below their respective AIs (7.5 µg/d, 10 mgα−TE, 500 mg/d, and 2000 mg/d, respectively). The mean magnesium intake was also lower than the recommended magnesium intake (RNI: 225 mg/d).

Other nutrient intakes were compared to the estimated average requirement (EAR) and/or tolerable upper intake level (UL).

### 3.4. Food Intake

**Figure 2:**
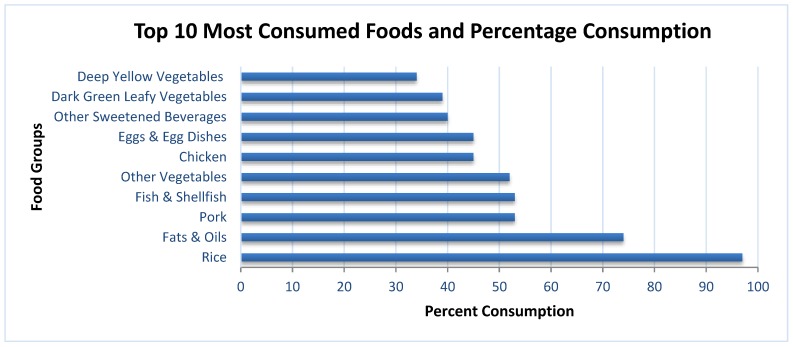
Percentage of participants consuming each food group based on a 24 h recall.

Refined rice, fats and oil, pork, fish, and other vegetables were the top five foods most consumed. The next five food items were chicken, egg and egg dishes, other sweetened beverages, dark-green leafy vegetables, and deep-yellow vegetables. The top five food sources of energy were rice, pork, fats and oils (mostly derived from plant sources), chicken, and bread. Half of the total carbohydrates came solely from refined rice, followed by bread, other sweetened beverages, noodles, and soft drinks. Refined rice, pork, fish, chicken, and egg and egg dishes were the top five contributors of protein. Forty percent (40%) of the total fat intake came from pork, while the other food sources were fats and oil (mostly plant sources), chicken, sausages, and egg (Figure 2).

Pork, refined rice, bread, noodles, and chicken were the top five food sources of thiamin. For riboflavin, pork, egg, chicken, fish, and powdered milk were the top contributors. Pork, chicken, deep-yellow vegetables, dark-green leafy vegetables, and fish were the top five food sources of vitamin A. Although almost half (42%) of the total vitamin C came from fruits and dark-green leafy vegetables, the prevalence of inadequate levels of vitamin C was still high. Bread, other vegetables, beans, nuts. and peas, dark-green leafy vegetables, and egg were the top five contributors of folate. Although iron and calcium content is not high in rice, it turned out that rice was the top contributor of iron, and the second highest for calcium. The other food sources of iron were pork, bread, sausages, and chicken, whereas for calcium, it was fish, chicken, powdered milk, and pork.

**Figure 3:**
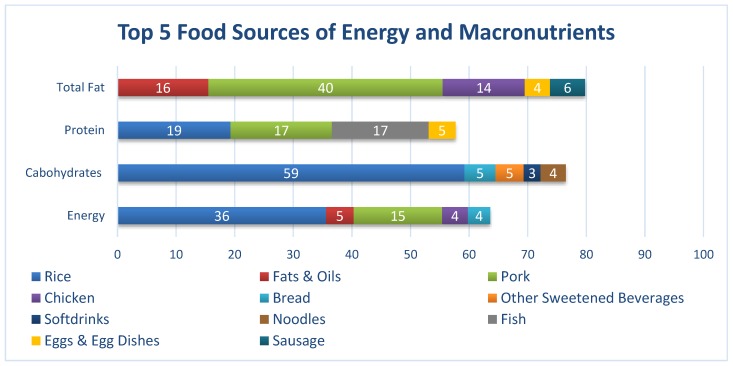
Percentage contributions of each food groups to the total energy, carbohydrate, protein, and total fat intake.

As seen in Figure 3, with regard to total energy intake, Filipino working adults consumed a significantly higher percentage of rice (36%) and pork (15%) and significantly lower percentages of fats and oils (5%), chicken (4%), as well as bread (4%). In terms of carbohydrates, rice (59%) had the highest percentage contribution, followed by bread (5%), other sweetened beverages (5%), noodles (4%), and soft drinks (3%). Contributions to the total fat intake were made mainly by the pork group (40%), followed by fats and oils (16%), chicken (14%), sausages (6%), and egg and egg dishes (4%). Lastly, with respect to protein intake, the rice group (19%) had the highest percentage contribution, preceded by pork (17%), fish (17%), and egg and egg dishes (5%).

**Figure 4:**
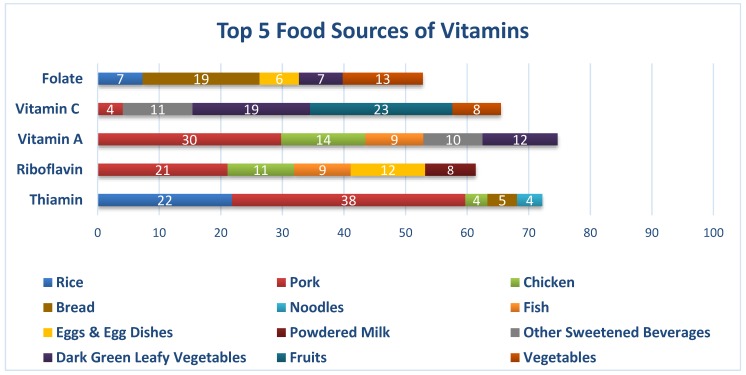
Percentage contributions of each food group to the vitamin intake of Filipino working adults.

As seen in Figure 4, pork, refined rice, bread, noodles, and chicken were the top five food sources of thiamin. For riboflavin, pork, egg, chicken, fish, ye and powdered milk were the top contributors. Pork, chicken, deep-yellow vegetables, dark-green leafy vegetables, and fish were the top five food sources of vitamin A. Although almost half (42%) of the total vitamin C came from fruits and dark-green leafy vegetables. the prevalence of inadequate vitamin C was still high. Bread, other vegetables, beans, nuts, and peas, dark-green leafy vegetables, and egg were the top five contributors of folate.

**Figure 5:**
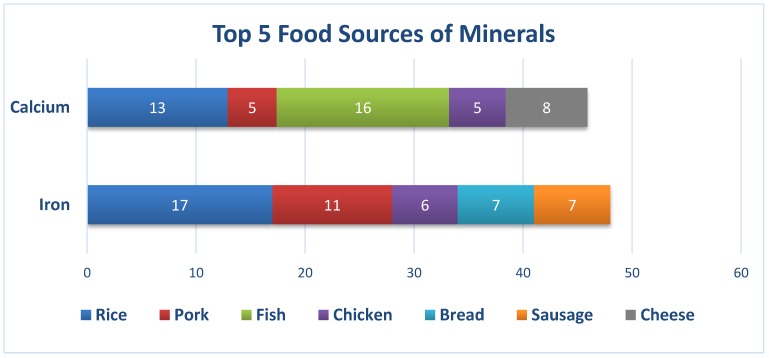
Percentage contributions of each food group to the calcium and iron intake of Filipino working adults.

As seen in Figure 5, fish, refined rice, cheese, pork, and chicken were the top five food sources of calcium, while iron mainly came from refined rice, pork, sausage, bread, and chicken in the usual diet of Filipino working adults. Although calcium can be found in large amounts in beans and lentils, seeds, fish (e.g., salmon and sardines), and green leafy vegetables, the diets of working adults seemed to lack these food items.

### 3.5. Physical Activity

**Figure 6:**
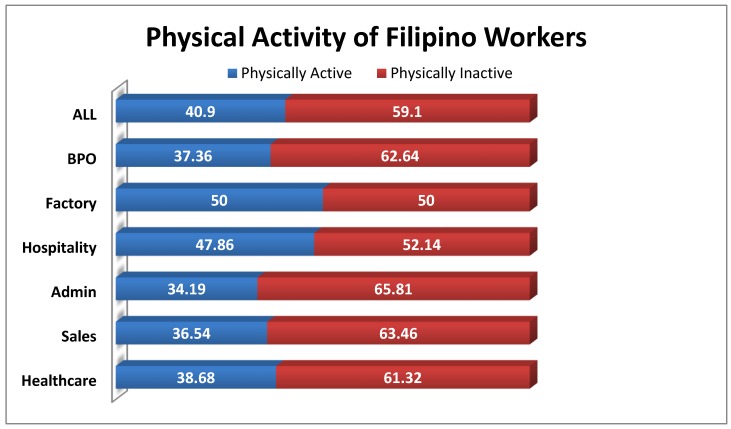
Percentage distribution of the respondents’ physical activity by sector.

Almost 6 in 10 respondents were found to be physically inactive (59.10%). The highest percentage of physical inactivity was seen in the admin sector (65.81%). In fact, a majority of the respondents per sector were physically inactive, except from the factory sector which had about 50% of physically inactive respondents (Figure 6).

### 3.6. Medical History

It was revealed that hypertension (25.51%) and acute infection (25.51%) were the most common conditions that were currently being treated among the respondents, while maintenance/prescription drugs (42.06%) and vitamins and mineral supplements (39.56%) were mostly taken. For the past year, reasons for the hospitalization of the respondents were mostly due to infections (33.08%) and chronic diseases (24.06%). Lastly, hypertension (21.50%) and diabetes (20%) were the most common diseases present in their families (Table 5).

## 4. Discussion

This study evaluated the usual nutrient intakes and food sources of working adults in the Philippines, where the employment rate of those who are 15 years old and over is about 94.3%. About 56.3% are in the Services sector, and 25.4% and 18.3% are in the Agriculture and Industry sectors, respectively [16]. Developing nations like the Philippines need to break the cycle of poor nutrition, low productivity, and low wages. Generally, lack of access to healthy foods has been cited as one of the main barriers to a healthy diet in the workplace.

### 4.1. Inadequate Energy and Nutrient Intake

The results of the study indicated key nutrients among the working adults. It has been postulated that a poor diet for working adults, resulting in either under-nutrition or overweight and obesity, is causing up to a 20% loss in productivity [14]. Data on the usual energy and nutrient intakes of the respondents from a 2-day, non-consecutive, 24 h food recall showed that the mean usual energy intake of the respondents did not meet the estimated energy requirement (EER), but looking closely at the percentage contribution of the different macronutrients to total energy intake, the protein and total fat intake as a percentage of total energy intake was 58% and 34% excessive, respectively. Protein mainly came from rice (19%), pork (17%), and fish (17%), while the fat intake was derived from pork (40%). From this, it can be deduced that the prime source of energy came from proteins and fats. Studies have strongly associated excessive fat and protein consumption with increasing BMI among adults [25], which was seen in this present study, wherein a BMI >25 was 47%. Other studies associated an increase of about 23% to risk in early death among males where their primary source of protein came from animals, compared to their plant-based protein-equivalent group [26]. However, it must still be noted that in general, protein consumption among working adults does not meet the RNI. The top three food groups consumed every day by all the respondents during the reference period of the past 7 days were refined rice (97%), oils and fats (74%), and pork (53%). Top food sources for the energy of working adults were rice (35.6%), pork (15.1%), fats and oils (4.7%), chicken (4.4%), and bread (3.8%). This may explain the excessive protein and fat intake and inadequate fiber intake of only 30% to 40% of the WHO Recommended Nutrient Intake (RNI) since the top foods consumed are rich in protein and fats but lack dietary fiber. The fat intake of the respondents had more saturated fats (pork = 40%), which are known for their loe density lipoprotein (LDL) cholesterol-raising potential and effects on risks of cardiovascular disease (CVD) [27,28]. Excess dietary saturated fat intake can be stored in the body, and when not expended, can accentuate risks of obesity [29]. It is also noted that carbohydrate intake as a percentage of total energy among all sectors was inadequate, but still represented about 47.7% of the total energy intake. Top sources for carbohydrates of the working adult population came from rice (35.6%), bread (5.3%), sweetened beverages (4.8%), and noodles (4.3%), which are considered “low-quality carbohydrates” because of their poor nutrient content and association with risks of disease [30]. Similar consumption patterns in the USA involving sugar-sweetened beverages were most likely associated with higher energy consumption, contributing to the obesity epidemic [31]. Legislation on heavily taxed sugar sweetened beverages was implemented in the Philippines early in 2018, and health incomes of this tax have yet to be evaluated [32].

The poor quality of food intake of respondents is reflected in the high prevalence of nutrient inadequacies: iron (99%), folate (98%), riboflavin (96%), calcium (94%), vitamin C (87%), thiamin (76%), and vitamin A (40%). This strongly suggests that consumption of energy-dense foods by the working adult population displaces the consumption of nutrient-dense foods [33]. This might be one factor that may have contributed to the high prevalence of hypertension (25.5%) and diabetes 6.6% among the respondents. In a similar study in the United States, subjects that tend to consume energy-dense, but nutrient-poor foods were found to consume an increased amount of sugars, fats, sodium, and alcohol and had a decreased diet quality, which increased the risk for these individuals to acquire chronic diseases, such as coronary heart disease (CHD) [34]. Other studies suggest that increased urbanization, coupled with globalization of the food market can be attributed to the dietary shift towards refined sugars, fats and oils, and processed meats, and a decline in fruit and vegetable consumption [35]. Multiple-micronutrient deficiency has been called the “hidden hunger”, as these nutrients can aggravate factors for an individual’s health outcome and quality of life [36]. Several studies have linked early-onset micronutrient deficiencies to an increased risk of chronic, non-communicable diseases later on in life, and thus, prevailing inadequate intakes of key micronutrients of working adults exposes this population to the development of NCDs. Poor nutrition among workers is already viewed as an occupational health hazard [37,38]. Micronutrient deficiency has a direct impact on worker productivity and performance, as pointed out in the study of Lukaski in 2004 [39,40]. In this study, infections, anemia, and CVD were mentioned as the pre-disposing health conditions of the respondents, and most were under maintenance medications (Table 5). Extremely high micronutrient inadequacies in iron (99% inadequacy), calcium (94% inadequacy), folate (98% inadequacy), and riboflavin (96% inadequacy) may predispose this population to higher risks of infections and NCDs.

In previous studies, workplace factors that were linked to improved eating habits of workers included food-related policies, health and nutrition promotion, creating a supportive nutrition-promoting environment, and offering healthy foods in the workplace canteen. Cross-level interactions between the workplace nutrition environment and canteen management attitude towards the health and diet of workers were significantly associated with eating practices [40]. Therefore, worksites are potential environments to promote healthy eating by making healthy food choices available in the canteens and neighborhood food establishments.

### 4.2. Physical Activity and Inactivity

This study indicated a high prevalence of physical inactivity among Filipino workers, with 59.1% of adults being classified as inactive since they did not meet the standard guidelines of the WHO global approach to physical activity and health [41]. Marked inactivity was predominant among the Admin sector, with 65.81% of adults not meeting the recommendation for physical activity, while factory workers had a half-on-half situation, citing 50% of its workers as active and the other half as inactive. Our data confirms the rising level of physical inactivity among adults aged 20 years and over cited in the 2013 National Nutrition Survey [11]. The literature has pointed out the detrimental effects of prolonged physical inactivity on people’s health [42,43]. Evidential research has stated that prolonged inactivity increases the risk for coronary vascular accidents, type II diabetes, cancer, obesity, musculoskeletal problems, and psychological disorders, including depression and anxiety [44]. The nature of administrative work done in a seated manner forcibly engages the worker to become inactive, where office work and driving tasks are especially characterized by long periods of uninterrupted sitting. As the number of administrative and desk jobs increase, the risk of an increasing prevalence of NCDs is imminent [45]. Research still continues to support the public health need of a culture suitable for regular physical activity.

This study highlighted that Filipino workers are vulnerable to disproportional macronutrient and micronutrient intake. Energy, carbohydrates, proteins, and key micronutrient intake were markedly inadequate. However, the percentage contribution of proteins and fats of the total energy consumption of working adults were found to be excessive. The high prevalence of overweight and obesity coupled with micronutrient deficiencies predispose these individuals to CVD and other NCDs. This is aggravated by a dietary pattern shift to an increasing intake of non-nutrient-dense foods, like refined sugars, fats and oils, and processed meat products, while having a diminishing intake of fruits and vegetables, coupled with the sedentary nature of their jobs.

These findings could be used by policy-makers in the Philippines as one of the bases for developing programs, interventional policies, and initiatives addressing the needs of these sectors, like the provision of healthy and safe food canteens in the workplace and its food environment.

### 4.3. Limitations of the Study

The study had a cross-sectional design; hence, there was no cause–effect relationship. Although the findings on intake is revealing and previous literature reviews support the negative consequences of high-fat, high-protein intake, we still consider the possibility of recall bias on food intake and physical activity data. Measurement of obesity was done only using BMI, and other indices, like the waist-to-hip ratio, or hip and waist circumference were not measured.

## Figures and Tables

**Table 1:** Calculated sample size by job sector.

Work Sector	Type of Work	Mean Age (y)	Calculated Sample Size
BPO ^1^	Call Center Agents	31	174 (14%)
Factory	Automotive, Industrial Plants	35	312 (25%)
Hospitality and Foods	Hotel staff, Food Service workers, Waiters, Cooks	32	140 (11%)
Administration	Office Work	33	272 (21%)
Sales	Sales Agent and Associates (Real estate, Malls etc.)	30	260 (21%)
Healthcare	Nurses, Hospital Staff	32	106 (8%)
Total sample (*n*)			1264

^1^ Abbreviations: BPO – Business Process Outsourcing.

**Table 2:** Characteristics of the participants (*n* = 1264).

Socio-Economic, Demographic Characteristics and Nutritional Status			
		*n*	%
**Gender**	Male	631	49.9
	Female	633	50.1
**Civil Status**	Single	754	59.6
	Married	469	37.1
	Separated/Widowed	41	3.24
**Educational Status**	No education	2	0.2
	Elementary graduate	75	5.9
	High school graduate	390	30.8
	Vocational/College graduate	797	63
**Job Sector**	BPO ^1^	174	13.8
	Factory	312	24.7
	Hospitality	140	11.1
	Admin	272	21.5
	Sales	260	20.6
	Healthcare	106	8.4
**Household Monthly Income**	Less than Php25,000	404	32
	Php25,000-Php75,000	443	35
	Php75,001- Php150,000	136	10.8
	More than Php150,000	89	7
	Don’t know/Decline to respond	192	15.3
**Sources of Funds (multiple answer)**	Salary/Wages	1264	100
	Selling goods/services	244	19.3
	Money Lending/Others	40	3.2
**Tenure in Work**	Less than a year	395	31.2
	1 to 5 years	516	40.8
	5 years or more	353	27.9
**Nutritional Status and Physical Measurements**			
**Nutritional Status**	Underweight	50	4
	Normal	618	48.9
	Overweight	391	30.9
	Obese	205	15.8
**Physical Measurements (Mean ± SD)**	Body Weight (kg)	65.1	15.2
	Height (cm)	159.8	8.6
	BMI (kg/m^2^)	25.4	4.7

^1^ Abbreviations: BPO: Business Process Outsourcing; SD: standard deviation.

**Table 3:** Mean energy and nutrient intake of working adults by sector.

	Mean Intake ± SE						
Nutrients	All (*n* = 1264)	BPO (*n* = 174)	Factory (*n* = 312)	Hospitality (*n* = 140)	Admin (*n* = 272)	Sales (*n* = 260)	Healthcare (*n* = 106)
Energy(kcal/d)	1768.8 ± 13	1761.3 ± 31	1715.3 ± 25.3	1990.5 ± 48.8	1806.9 ± 31.7	1745.3 ± 28	1610.6 ± 38.6
EER ^G^ (kcal)	2401.5 ± 12	2474.4 ± 37	2440.8 ± 21.3	2444.7 ± 36.5	2333.3 ± 26.4	2382.4 ± 27	2335.2 ± 45.3
**Macronutrients**							
Total fat (g/d)	56.56 ± 0.5	61.6 ± 1.3	47.2 ± 0.9	65.1 ± 2	58.6 ± 1.1	58.7 ± 1.0	57 ± 1.4
Saturated fat (g/d)	26.73 ± 0.3	29.8 ± 0.7	23.3 ± 0.6	29.6 ± 0.8	26.9 ± 0.7	27 ± 0.6	26.5 ± 0.5
MUFA (g/d)	21.20 ± 0.2	23.9 ± 0.6	16.9 ± 0.4	24.7 ± 0.7	22.1 ± 0.5	22.2 ± 0.5	20.1 ± 0.5
	9.45 ± 0.1	10.2 ± 0.3	7.9 ± 0.2	10.7 ± 0.4	10.2 ± 0.2	9.8 ± 0.2	8.9 ± 0.3
PUFA (g/d)							
Carbohydrate (g/d)	238.47 ± 2	216.3 ± 4.1	251.4 ± 4	268.5 ± 7.5	245.1 ± 4.7	229.5 ± 4.1	203.4 ± 5.7
Total sugars (g/d)	31.13 ± 0.5	33.4 ± 1.2	27.6 ± 0.8	34.4 ± 1.5	33.4 ± 1	29.3 ± 1.2	32.6 ± 1.4
Dietary fiber (g/d)	7.97 ± 0.07	7.5 ± 0.2	8 ± 0.1	8.4 ± 0.2	8.6 ± 0.2	7.8 ± 0.2	7.2 ± 0.3
Protein (g/d)	67.14 ± 0.5	67.9 ± 1.5	64.6 ± 1.1	76.6 ± 1.6	66.1 ± 1.2	67.2 ± 1.2	63.7 ± 1.6
**As percentage of Total Energy**							
Total fat (%)	27.75 ± 0.2	30.1 ± 0.5	23.6 ± 0.3	28.2 ± 0.5	28.6 ± 0.3	28.9 ± 0.3	30.5 ± 0.4
Protein (%)	15.5 ± 0.05	15.6 ± 0.08	15.3 ± 0.1	15.9 ± 0.2	15 ± 0.09	15.6 ± 0.1	16.5 ± 0.2
Carbohydrate (%)	55.27 ± 0.2	51.9 ± 0.5	59.8 ± 0.4	54.7 ± 0.6	55 ± 0.3	54.1 ± 0.4	51.6 ± 0.6
**Antioxidants**							
Vitamin C (mg/d)	33.24 ± 0.6	34 ± 1.4	30.1 ± 0.9	29.1 ± 1.4	35.7 ± 1.3	36 ± 2	36.9 ± 2.6
Vitamin E (mgα-TE)	4.42 ± 0.04	4.6 ± 0.07	4.1 ± 0.06	4.7 ± 0.1	4.6 ± 0.08	4.3 ± 0.1	4.6 ± 0.2
**B vitamins**							
Thiamin (mg/d)	0.77 ± 7.4	0.8 ± 0.01	0.7 ± 0.01	0.9 ± 0.02	0.8 ± 0.02	0.8 ± 0.02	0.7 ± 0.02
Riboflavin (mg/d)	0.61 ± 5.8	0.6 ± 0.01	0.6 ± 0.01	0.7 ± 0.02	0.6 ± 0.01	0.6 ± 0.01	0.6 ± 0.02
Niacin (mg/d)	21.01 ± 0.2	21.2 ± 0.5	21.2 ± 0.3	24 ± 0.5	20.1 ± 0.4	20.7 ± 0.4	19.6 ± 0.5
Vitamin B6 (mg/d)	2.42 ± 0.03	2.4 ± 0.06	2.3 ± 0.05	2.5 ± 0.05	2.4 ± 0.07	2.7 ± 0.09	2.1 ± 0.07
Vitamin B12 (µg/d)	4.41 ± 0.06	4.1 ± 0.2	4.7 ± 0.1	4.9 ± 0.2	4 ± 0.1	4.4 ± 0.1	5.1 ± 0.3
Folate (µg DFE ^E^)	179.58 ± 1.7	176 ± 5.1	187.3 ± 3.3	164.6 ± 4.9	184.3 ± 2.9	180 ± 3.6	170.8 ± 6.5
**Bone-related nutrients**							
Calcium (mg/d)	354.85 ± 3.7	339.3 ± 5.6	348.8 ± 7.8	399.4 ± 14.4	371 ± 8.5	334.4 ± 9.5	373.6 ± 15.3
Phosphorus (mg/d)	911.42 ± 7.1	883.7 ± 16.8	911.7 ± 14.9	1015.5 ± 19.7	909.4 ± 16.5	898.6 ± 16.6	859.3 ± 21.7
Magnesium (mg/d)	183.52 ± 1.5	190.0 ± 3.9	178.1 ± 2.8	198.2 ± 4.6	188.1 ± 3.3	178.3 ± 3.1	169.3 ± 5.5
Vitamin D (µg/d)	4.0 ± 0.03	3.5 ± 0.08	4.4 ± 0.07	4.4 ± 0.1	3.7 ± 0.08	4 ± 0.09	4 ± 0.1
**Other micronutrients**							
Vitamin A (μgRE/d)	567.46 ± 7.1	557.4 ± 20.3	531.2 ± 13.4	731 ± 24.6	610.4 ± 14.2	503.2 ± 11.5	528.5 ± 26.3
Iron (mg/d)	9.71 ± 0.08	9.6 ± 0.2	9.4 ± 0.1	10.7 ± 0.3	9.9 ± 0.2	9.6 ± 0.2	9.2 ± 0.3
Zinc (mg/d)	9.71 ± 0.06	7.4 ± 0.2	6.7 ± 0.1	8.2 ± 0.2	7.5 ± 0.1	7.5 ± 0.1	7 ± 0.2
Sodium (mg/d)	1038.6 ± 13	1160.1 ± 33	916.9 ± 21.6	1086.9 ± 48.4	1096.1 ± 31.9	1076.8 ± 29	971.6 ± 35.4
Potassium (mg/d)	1617.8 ± 12	1617.7 ± 29	1532.4 ± 24.5	1833.1 ± 36.2	1670.6 ± 28.1	1598.7 ± 27	1507.2 ± 37.1
Selenium (µg/d)	116.84 ± 1	116.7 ± 2.2	111.2 ± 1.8	139.8 ± 3	113.9 ± 2.2	117.2 ± 2.1	110.8 ± 3.3

*SE*–standard error.

**Table 4:** Prevalence of inadequate and excessive intake of nutrients by sector.

	Prevalence of Inadequacy (% < EAR ^A^/LAMDR ^B^)							Prevalence of Excessiveness (% > UL ^C^/UAMDR ^D^)						
	All (*n* = 1264)	BPO (*n* = 174)	Factory (*n* = 312)	Hospitality (*n* = 140)	Admin (*n* = 272)	Sales (*n* = 260)	Healthcare (*n* = 106)	All (*n* = 1264)	BPO (*n* = 174)	Factory (*n* = 312)	Hospitality (*n* = 140)	Admin (*n* = 272)	Sales (*n* = 260)	Healthcare (*n* = 106)
**Macronutrients**														
Protein	23.9	22.8	29.1	8.4	26.5	23.9	28.3	-	-	-	-	-	-	-
**As percentage of Total Energy**														
Total Fat (%)	<1	<1	5.9	1.2	0	<1	0	33.7	49.6	13.8	37.7	36.3	39.2	54.1
Protein (%)	0	0	<1	0	0	0	<1	58.1	72.7	51.7	64.1	47.5	61.5	70.8
Carbohydrate (%)	47.7	67.3	24.7	51.1	49.5	55.2	69.8	<1	<1	1.2	<1	0	0	0
**Antioxidants**														
Vitamin C	87.1	88.8	92.6	92.7	85.2	81.6	82.5	0	0	0	0	0	0	0
Vitamin E	-	-	-	-	-	-	-	0	0	0	0	0	0	0
**B Vitamins**														
Thiamin	76.6	83.8	86.2	63.8	72.9	71.2	84.9	-	-	-	-	-	-	-
Riboflavin	95.8	98.9	96.1	90.8	93.9	95.3	93.6	-	-	-	-	-	-	-
Niacin	3.1	4.6	3.6	<1	4.4	2.7	2.7	2.3	3.1	2.9	4.7	2.1	1.7	<1
Vitamin B6	8.7	5.9	11.2	<1	10.6	11.1	10	0	0	0	0	0	0	0
Vitamin B12	6.2	11.8	6.5	3.3	11.6	2.1	6.3	-	-	-	-	-	-	-
Folate	97.9	96.9	97.5	98.6	99.1	97.9	97.1	0	0	0	0	0	0	0
**Bone-Related Nutrients**														
Calcium	94.7	99.8	94.2	88.6	93.2	94	91.6	0	0	0	0	0	0	0
Phosphorus	7.5	6.9	8.4	1.3	8.8	9.8	8.4	0	0	0	0	0	0	0
Magnesium	-	-	-	-	-	-	-	-	<1	<1	<1	<1	<1	<1
Vitamin D	-	-	-	-	-	-	-	0	0	0	0	0	0	0
**Other Micronutrients**														
Vitamin A	39.7	43.4	45.6	16.7	29.4	48.1	48.8	0	0	0	0	0	0	0
Iron	99	99.4	99.3	96.6	98.5	99.5	98.6	0	0	0	0	0	0	0
Zinc	2.5	2.2	6.2	<1	2.1	1.7	1.8	0	0	0	0	0	0	0
Selenium	0	0	0	0	0	0	0	0	0	0	0	0	0	0

^A^ Estimated Average Requirements; ^B^ Lower Acceptable Macronutrients Distribution Range; ^C^ Tolerable upper intake level or upper limits per day; ^D^ Upper Acceptable Macronutrients Distribution Range.

**Table 5:** Percentage distribution of the respondents with medical conditions, and medications taken.

Disease/Medical Condition Currently Being Treated ^m^	All (*n* = 1264)	
	*n*	%
Acute infection	62	25.51
Allergy	9	3.70
Anemia	6	2.47
Cancer	3	1.23
Cardiovascular disease	5	2.06
Digestive & liver problem	21	8.64
Diabetes	16	6.58
Electrolyte imbalance	17	7.00
Gout, arthritis	11	4.53
Hypertension	62	25.51
Kidney disease	3	1.23
Muscle/body pain	18	7.41
Neurological disorder	5	2.06
Pulmonary disorder	26	10.70
Others (HIV, genetic diseases)	3	1.23
**Medications/Non-Prescription Drugs or Herbal Supplements Presently Taken ^m^**		
Herbal/food supplements	71	22.12
Hormonal/birth control pills	15	4.67
Maintenance/prescription drugs	135	42.06
Slimming pills	2	0.62
Vitamin & mineral supplements	127	39.56

^m^ Multiple answers.

## Abbreviations

WHO	World Health Organization
EAR	Estimated Adequate Requirement
AMDR	Acceptable Macronutrient Distribution Ranges
EER	Estimated Energy Requirements
GDP	Gross Domestic Product
LMIC	Low-Middle Income Countries
NCD	Non-Communicable Diseases
COPD	Chronic Obstructive Pulmonary Disease
FCT	Food Composition Table
BPO	Business Process Outsourcing
BMI	Body Mass Index
IOM	Institute of Medicine
PAL	Physical Activity Level
AI	Adequate Intake
RNI	Recommended Nutrient Intakes
FNRI	Food and Nutrition Research Institute
CIOMS	Council for International Organizations of Medical Sciences
FIERC	FNRI Institutional Ethics Review Committee
ICF	Informed Consent Form
BMR	Basal Metabolic Rate
LDL	Low Density Lipoprotein
CVD	Cardiovascular Disease
CHD	Coronary Heart Disease
